# Antibiotic consumption in the public sector of the Limpopo province, South Africa, 2014–2018

**DOI:** 10.4102/sajid.v37i1.462

**Published:** 2022-10-25

**Authors:** Tiyani C. Mthombeni, Johanita R. Burger, Martha S. Lubbe, Marlene Julyan

**Affiliations:** 1 Department of Medicine Usage in South Africa (MUSA), Faculty of Health Sciences, North-West University, Potchefstroom, South Africa

**Keywords:** pharmaceutical sales data, antibiotic consumption, antibiotic utilisation, Anatomical Therapeutic Chemical, defined daily dose, ATC DDD per 1000 inhabitants per day, 75% drug utilisation index, Limpopo, South Africa

## Abstract

**Background:**

Antibiotic consumption (ABC) surveillance is a critical component of the strategic priority response to the increasing antibiotic resistance threat. Levels of ABC at the national and provincial levels in South Africa are unknown because of inadequate ABC surveillance systems and literature. Antibiotic consumption in the public sector of Limpopo province, South Africa, 2014-2018

**Methods:**

This retrospective study used sales data retrieved from a pharmaceutical warehouse distribution database to quantify ABC. Antibiotic consumption was measured by the defined daily dose (DDD) per 1000 inhabitants per day (DID) and 75% drug utilisation index (DU75%). Change in consumption was measured by the compound annual growth rate (CAGR).

**Results:**

Between 2014 and 2018, the mean ABC was 4.6 ± 1.0 DID, with an overall decrease of 1.6% in the CAGR. Penicillins (2.3 ± 0.8; 50.0%), sulphonamide and trimethoprim combinations (1.4 ± 0.3 DID; 30.4%) were the most consumed antibiotics. Macrolides had the highest relative increase in consumption during the study period, with a CAGR of 18.5%. In contrast, tetracyclines had the highest relative decrease in consumption, with a CAGR of 100.0%. The CAGR ratio for broad- to narrow-spectrum increased by 39.3%, from 0.4 in 2014 to 2.1 in 2018. The DU75% comprised amoxicillin (28.4%), sulphamethoxazole and trimethoprim (SMX-TMP) (27.2%), doxycycline (12.3%) and azithromycin (9.2%).

**Conclusion:**

While ABC remained relatively stable throughout the study, there was an increase in broad-spectrum ABC that requires further investigation.

**Contribution:**

This study contributes ABC surveillance data in Southern Africa, described by ATC classification, which is essential for monitoring and evaluating antibiotic stewardship programmes.

## Introduction

Antibiotic resistance (ABR) is a natural development process for bacterial organisms and remains one of the leading concerns in global health.^1^ The global burden of diseases related to ABR is projected to have contributed to 4.95 million mortalities in 2019, with 1.27m deaths directly ascribed to ABR.^2^ The development of ABR is accelerated by the inappropriate and increased consumption of antibiotic drugs.^3,4^ Globally, antibiotic consumption (ABC) increased by 46%, from 9.8 defined daily doses (DDDs) per 1000 inhabitants per day (DID) in 2000 to 14.3 DID in 2018.^3^ Antibiotic resistance is a global concern that necessitates a coordinated international, national and local response.^2^

The World Health Organization (WHO) has identified scaling up ABC surveillance as a critical strategic priority for monitoring ABR burden and global ABR response.^5^ The 2015 World Health Assembly resolved that member states must begin tracking ABC as part of a worldwide action plan to address the growing public health threat posed by ABR, which the United Nations General Assembly later endorsed.^1^ Similarly, the United States (US) Centers for Disease Control and Prevention recognised that ABC surveillance is a core component of antimicrobial stewardship programmes (ASPs) because it enables tracking and monitoring of ABC.^6^ Antibiotic consumption surveillance can be achieved by analysing ABC data, which are estimated antibiotic usage data derived from accumulated data sources such as import or wholesaler or health insurance databases, without patient-level details or justifications for use.^7^ Therefore, the availability of ABC data is a critical requirement to support coordinated global, national and local responses to the ABR threat.

Antibiotic consumption surveillance data are predominately limited to high-income countries, as there is an underrepresentation of surveillance systems and data from low- to middle-income countries (LMICs) in global studies.^3^ Therefore, there is a persistent lack of surveillance data on ABC from LMICs.^3,4^ Furthermore, there is a paucity of research on ABC in sub-Saharan Africa (SSA) at regional, national or provincial levels.^8,9,10,11^ Despite rising ABC in LMICs, central SSA had the lowest ABC per capita (8.2 DID) in 2018 and has not increased much since 2000.^3^ Sub-Saharan Africa was underrepresented in early ABC reports on a global level.^3,4,12^

There are variations and heterogeneity in the total ABC and antibiotic classes across countries, provincial contexts and the healthcare sector.^3^ Moreover, provincial estimates indicate differences in ABC within countries, highlighting underlying healthcare access challenges, particularly among the poor and rural populations.^3^ The South African healthcare system is divided into the state-financed public sector (73.0%) and private sector (27.0%), reimbursed by medical aid schemes or individual out-of-pocket payments.^13^ The majority of rural South Africans rely on public sector facilities as their initial point of contact for healthcare services.^14^ Compared with other LMICs, South Africans’ access to registered pharmaceuticals in the public sector is severely restricted.^14^ The Limpopo province comprises approximately 80.0% of rural settlements and impoverished communities that rely heavily on public sector health institutions (84.0%) for health services, with only 9.9% of the province’s 5.9m residents with medical insurance.^13^

According to the 2020 Global Antimicrobial Resistance and Use Surveillance (GLASS) report, South Africa has not implemented a surveillance system for ABC on both the national and provincial levels.^15^ In low-resource settings, determining ABC, monitoring and evaluating AMS policy and activities are challenging because of a lack of surveillance systems and data.^16^ Therefore, this study aimed to quantify and describe ABC retrospectively over five years (2014–2018) in the public sector of the Limpopo province, South Africa.

## Methods

### Study design

A retrospective, descriptive and repeated cross-sectional study design was implemented using aggregated sales data from a central, provincial public health pharmaceutical warehouse.

### Study settings

The study was conducted in the Limpopo province in South Africa. The province is located in the northern region of South Africa, sharing a border with three SSA countries (Botswana, Mozambique and Zimbabwe).^17^ The LPPD, where the data for this study were collected, is administered by the Provincial Department of Health (PDoH) and is located within the Capricorn district municipality in the Limpopo province. The PDoH provides healthcare services throughout the province through its 30 district hospitals, five regional hospitals, four specialised hospitals, two tertiary hospitals, 481 community health centres and fixed clinics and 42 mobile clinics.^18^ The LPPD is responsible for the direct and indirect distribution of pharmaceuticals, including antibiotics, to all district and specialised hospitals and fixed and mobile primary healthcare institutions. Furthermore, tertiary and regional hospitals utilise a direct procurement approach, supplemented by the LPPD on an emergency basis.^18^

### Study inclusion and exclusion criteria

This study focused on the J01 (antibiotic for systemic use) antimicrobial group of the Anatomical Therapeutic Chemical (ATC) classification system.^19^ Therefore, topical antibiotics, antimycotics and antifungals (J02), antivirals (J05), antimycobacterial (J04), antimalarials (P01B), antiprotozoals used as antibiotic agents (P01AB), antibiotics used as intestinal anti-infectives (A07AA) and antiseptic agents were excluded.^7,19^ Moreover, the authors focused on ABC for human consumption only; nonhuman use (e.g. agriculture for both plants and animals) was therefore excluded.

### Study data sources, variables and procedure

This study used sales data from the public sector central depot’s pharmaceutical distribution database data to quantify and describe antibiotic distribution trends retrospectively over five years (2014 to 2018).

The GLASS methodology was adopted for this study^7^; that is, the ATC classification system and the DDD were used as units of measurement.^19^ The ATC classification system divides drugs into distinct groups (named after letters in some alphabets) based on their mode of action and therapeutic and chemical characteristics.^7^ The active substances in the ATC system are classified according to the organ or system on which they act, as well as their therapeutic, pharmacological and chemical properties. Medications are categorised into five distinct levels. The DDD is the ‘assumed average daily maintenance dose for a drug used for its primary indications in adults’.^7^ The ATC and DDD are available as a downloadable computer software tool known as the WHO antimicrobial consumption tool.^20^ Data (provided as the total number of packages distributed per calendar year) were manually entered into the software to calculate the ABC.

### Reported metrics and data analysis

The results for this study are provided following selected ABC quality indicators. The total ABC and consumption by different antibiotic subgroups are reported in DID.^19^ The estimated total annual human population (covering both public and private healthcare sectors) of the Limpopo province available from Statistics South Africa was utilised for DID adjustments to account for population size variations and to enable comparison across time and population groups, because it was not probable to estimate the population of the public sector only.^12,13^ The relative consumption by the antibiotic subgroup is presented as a percentage of the total consumption of antibiotics for systemic use. To show changes in consumption over time, the total antibiotic or pharmacological subgroups or antibiotic agents at ATC level five, compound annual growth rate (CAGR) was determined.^21^ The broad- to narrow-spectrum ratio was calculated through the consumption of broad-spectrum penicillins, cephalosporins, macrolides (except erythromycin) and fluoroquinolones (broad-spectrum penicillins [J01CR] + broad-spectrum cephalosporins [J01DC + J01DD] + broad-spectrum macrolides [J01F] – erythromycin [FA01]) to the consumption of narrow-spectrum penicillins, cephalosporins and macrolides (narrow-spectrum penicillins [J01CE] + narrow-spectrum [J01DB] + erythromycin [FA01]).^21^ Arithmetic means were calculated to determine the central tendency of the ABC data. The total mean consumption of different antibiotic agents at ATC level five was ranked from highest to lowest, and the most commonly consumed antibiotic agents accounting for 75% of total mean consumption (DU75%) were established.^22^

### Ethical considerations

Ethical approval of the study was obtained from the North-West University Health Research Ethics Committee (reference number NWU-00312-20-A1). Data sources access permission was granted by the Head of the Limpopo Department of Health (LDoH) (reference number LP-202003-012) and a letter of goodwill was obtained from the manager of the Limpopo Province Pharmaceutical Depot (LPPD) before conducting the study.

## Results

### Overall antibiotic consumption density

The overall mean for antibiotic for systemic use (J01) consumption during the study period (from 2014 to 2018) was 4.6 ± 1.0 DID (Table 1). Between 2014 and 2018, total ABC decreased by a CAGR of 1.6%.

**TABLE 1:** Total antibiotic consumption and by route of administration.

Route of administration	2014	2015	2016	2017	2018	Mean	s.d.	% Contribution	CAGR %
Oral (DID)	4.00	4.60	4.20	6.30	3.80	4.60	± 1.0	98.9	−1.4
Parenteral (DID)	0.07	0.04	0.04	0.06	0.03	0.00	± 0.0	1.1	−14.0

**Total (ABC in DID)**	**4.10**	**4.70**	**4.20**	**6.30**	**3.80**	**4.60**	**± 1.0**	**100.0**	**-1.6**

Note: As a result of small numbers, the DIDs for parenteral are given to two points after the decimal.

s.d., standard deviation; CAGR, compound annual growth rate; DID, defined daily dose per 1000 inhabitants per day; ABC, antibiotic consumption.

### Consumption by route of administration

Orally administered antibiotics contributed 98.9%, compared with 1.1% for parenteral administered antibiotics (Table 1). The CAGR for parenteral administered systemic ABC decreased by 14.0% from 2014 to 2018.

### Absolute and relative antibiotic consumption density at Anatomical Therapeutic Chemical levels 3 and 4

**FIGURE 1:**
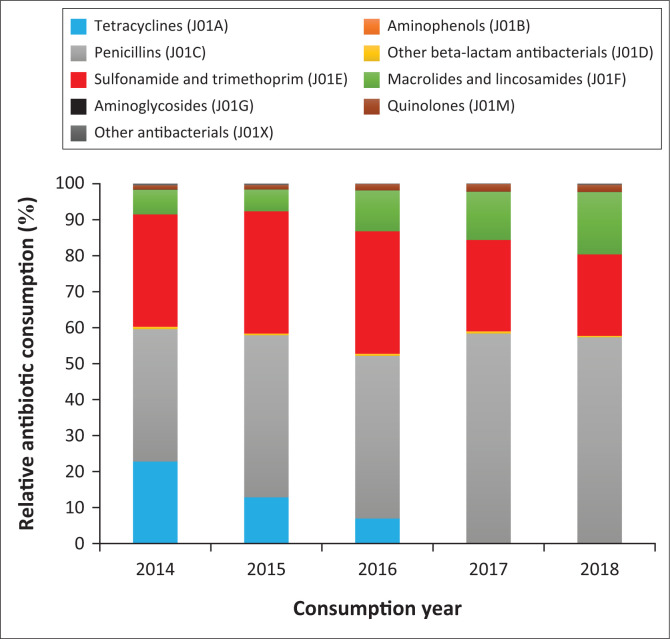
Relative antibiotic consumption at anatomical therapeutic chemical level 3.

The penicillins (J01C) were the most consumed antibiotic subgroup, increasing from 35.8% of the relative consumption in 2014 to 45.0% in both 2015 and 2016 to more than half (58.4% and 57.3%) of relative consumption in 2017 and 2018, respectively (Figure 1 and Table 2). Sulphonamide and trimethoprim (J01E) contributed nearly one-third (31.2%, 34.0% and 34.0%) of the relative consumption in 2014, 2015 and 2016, respectively, before declining in 2017 and 2018. Other beta-lactam antibiotic (J01D) consumption remained below 1% of the total consumption during the study. The macrolides and lincosamides (J01F) subgroup increased from 6.9% to more than double by 2018, contributing 17.4% of the total antibiotics distributed. The quinolones (J01M) subgroup contribution increased from 1.2% of the total consumption in 2014 to 2.0% by 2018.

**TABLE 2:** Absolute and relative antibiotic consumption at Anatomical Therapeutic Chemical levels 3 and 4, mean and compound annual growth rate.

ATC level	2014		2015		2016		2017		2018		Mean	s.d.	CAGR %
	DID	%	DID	%	DID	%	DID	%	DID	%			
**ATC Level 3 absolute and relative consumption**													
Tetracyclines (J01A)	0.90	22.8	0.6	12.8	0.3	7.0	0.0	0.0	0.0	0.0	0.4	± 0.4	−100.0
Aminophenols (J01B)	0.00	0.0	0.0	0.0	0.0	0.0	0.0	0.0	0.0	0.0	0.0	± 0.0	0.0
Penicillins (J01C)	1.50	36.8	2.1	45.1	1.9	45.2	3.7	58.4	2.2	57.3	2.3	± 0.8	7.6
Other beta-lactam antibacterial (J01D)	0.00	0.7	0.0	0.5	0.0	0.6	0.0	0.6	0.0	0.4	0.0	± 0.0	−12.9
Sulphonamide and trimethoprim (J01E)	1.30	31.2	1.6	34.0	1.4	34.0	1.6	25.4	0.9	22.6	1.4	± 0.3	−7.7
Macrolides and lincosamindes (J01F)	0.30	6.9	0.3	6.1	0.5	11.3	0.9	13.4	0.7	17.4	0.5	± 0.3	18.5
Aminoglycosides (J01G)	0.00	0.1	0.0	0.0	0.0	0.0	0.0	0.0	0.0	0.0	0.0	± 0.0	−19.5
Quinolones (J01M)	0.10	1.2	0.1	1.2	0.1	1.8	0.1	2.2	0.1	2.0	0.1	± 0.0	9.5
Other antibacterials (J01X)	0.00	0.4	0.0	0.4	0.0	0.1	0.0	0.1	0.0	0.3	0.0	± 0.0	−9.4
**ATC Level 4 absolute and relative consumption**													
Beta-lactamase sensitive penicillins (J01CE)	0.40	9.0	0.5	10.0	0.2	4.0	0.8	12.5	0.3	8.8	0.4	± 0.2	−2
Combination penicillins (J01CR)	0.10	2.7	0.2	3.2	0.2	3.8	0.3	4.9	0.3	6.6	0.2	± 0.1	17.8
Third and fourth generation cephalosporins (J01DD+DE)	0.03	0.7	0.0	0.5	0.0	0.6	0.0	0.6	0.0	0.3	0.0	± 0.0	−13.6
Fluoroquinolones (J01MA)	0.10	1.2	0.1	1.2	0.1	1.8	0.1	2.2	0.1	2.0	0.1	± 0.0	9.2
Cephalosporins (J01DB + J01DC + J01DD + J01DE)	0.00	0.7	0.0	0.5	0.0	0.8	0.0	0.6	0.0	0.4	0.0	± 0.0	−12.9

**Total DID**	**4.10**	**-**	**4.7**	**-**	**4.2**	**-**	**6.3**	**-**	**3.8**	**-**	**4.6**	**± 1.0**	**-1.6**

DID, defined daily dose per 1000 inhabitants per day; s.d., standard deviation; CAGR, compound annual growth rate; ATC, Anatomical Therapeutic Chemical classification.

At ATC level 4 (Table 2), the relative consumption of beta-lactamase-sensitive penicillins (J01CE) to total consumption fluctuated, peaking at its highest at 12.5% in 2017. The relative consumption of combination penicillins (J01CR) increased (CAGR of 17.8%) from 0.1 DID (2.7%) in 2014 to 0.3 DID (6.6%) of the total consumption by 2018. The relative consumption of cephalosporins fluctuated below 1% of the total consumption, reaching a high of 0.8% in 2016 and a low of 0.4% in 2018.

### Change in consumption at Anatomical Therapeutic Chemical level 3 and 4

At ATC level 3, the consumption of macrolides and lincosamides (J01F) increased by 18.5% (Table 2). Quinolones’ (J01M) consumption increased by 9.2% over the study period. Consumption of penicillins increased with a CAGR of 7.6%. Tetracyclines were not distributed in 2017 and 2018. There was also a decrease in consumption of other beta-lactam antibiotics, sulphonamide and trimethoprim, aminoglycosides and other antibiotic pharmacological subgroups with a CAGR of 12.9%, 7.7%, 19.5% and 9.4%, respectively (Table 2). At ATC level 4, consumption of combination penicillins (J01CR) increased with a CAGR of 17.8%. There was a 13.6% CAGR decrease in the consumption of third and fourth-generation cephalosporins (J01DD + DE).

### Broad- or narrow-spectrum consumption ratio

The consumption of broad-spectrum antibiotics increased with a CAGR of 28.5%, while the consumption of narrow-spectrum antibiotics decreased with a CAGR of 12.9% (Table 3). There was a 39.3% CAGR increase in the consumption ratio of broad-to-narrow spectrum antibiotics during the study period.

**TABLE 3:** Broad- and narrow-spectrum antibiotic consumption.

Antibiotic spectrum	2014	2015	2016	2017	2018	Mean	s.d.	CAGR %
Broad (J01(CR + DC + DD + (F-FA01))) in DID	0.2	0.3	0.4	0.8	0.7	0.5	± 0.2	28.5
Narrow (J01(CE + DB + FA01)) in DID	0.6	0.5	0.2	0.8	0.3	0.5	± 0.2	−12.9
Ratio	0.4	0.7	2.6	1.0	2.1	1.3	± 0.9	39.3

s.d., standard deviation; CAGR, compound annual growth rate; J01CR, broad-spectrum penicillins; J01DC, third-generation cephalosporins; J01DD, fourth-generation cephalosporins; J01F, broad-spectrum macrolides; J01FA01, erythromycin; J01CE, narrow-spectrum penicillins; J01DB, first-generation cephalosporins; DID, defined daily dose per 1000 inhabitants per day.

### DU75% and change in consumption at Anatomical Therapeutic Chemical level 5

Four antibiotic agents (amoxicillin, SMX-TMP, doxycycline and azithromycin) comprised the overall DU75% during the entire study period (2014–2018) (Table 1-A1). Amoxicillin was the most consumed antibiotic agent, followed by SMX-TMP, doxycycline and azithromycin. The most significant change in consumption was observed for cephalexin, with a CAGR of 133.7% between 2014 and 2018. Seven other antimicrobial agents experienced an increase in consumption (Table 1-A1). At the same time, 14 antibiotics had a CAGR of −100.0% because they were not distributed in 2018. Ten antibiotics experienced a decreased consumption (Table 1-A1).

## Discussion

This study quantified and described ABC from sales data obtained from a pharmaceutical warehouse distribution’s database using the ATC and DDD index and tracked ABC change using CAGR, broad-to-narrow spectrum index and DU75%. At 4.6 ±1.0 DID between 2014 and 2018, the overall mean consumption rate was stable and low compared with global (14.1 DID) and SSA regional (8.2 DID) rates.^3,4^ It should, however, be taken into account that the total annual population^13^ for the Limpopo province was utilised as the denominator in this investigation because it was not possible to determine the population for the public sector alone, whereas the sales data represented only the public sector. The ABC sales data furthermore excluded antibiotics procured directly from five regional and two tertiary hospitals and health facilities under other governmental departments, such as Correctional Services and Military Health. This study’s estimates may, consequently, be an underestimate of the ABC in the province. Nevertheless, these findings can serve as a baseline for future ABC quantification and monitoring in the Limpopo province.

During the study period, oral and parenteral formulations accounted for 98.9% and 1.1%, respectively, with parenteral formulations decreasing by 14.0% compared with a 1.4% decrease in oral formulations. The rationale for a notable decrease in parenteral formulations could be linked to a global shortage of injectable penicillin, such as ampicillin and benzylpenicillin^23^ and a national shortage of injectable cephalosporins such as ceftriaxone during the study period.^24^

Globally, penicillins are the most consumed antibiotics subgroup, accounting for 39.0% of all antibiotics consumed, followed by cephalosporins and quinolones.^4^ Amoxicillin, in particular, is the most consumed agent globally.^12^ The findings of this study showed that similar to global trends, penicillins were the most consumed antibiotics subgroup, with amoxicillin as the most consumed agent. Penicillins are indicated as first-choice agents for common respiratory infections in the South African Standard Treatment Guidelines (STGs) and Essential Medicines List, which is the reference for selecting and procuring medicines for the public health sector in South Africa.^25^ Moreover, in 2018, penicillins (amoxicillin with enzyme inhibitor and ampicillin) were the most commonly prescribed antibiotics for hospitalised patients in South Africa for skin and soft tissue infections, pneumonia and sepsis.^26,27^ Furthermore, the increase in relative consumption of broad-spectrum penicillins (particularly combination penicillins, i.e. amoxicillin with enzyme inhibitor), macrolides and quinolones, corresponding with a decreased consumption of narrow-spectrum penicillins, resulted in 39.3% CAGR increase in the broad-to-narrow spectrum ratio (Table 3). These findings are consistent with trends shown in a national report by the South African National Department of Health, where broad-spectrum antibiotics accounted for more than 80% of total procurement between 2015 and 2017, with the proportion of broad-to-narrow spectrum antibiotics increasing from 10% in 2015 to 20% in 2017.^28^ Considering that total ABC remained stable over a period of five years (2014–2018) in this study, the increased broad-to-narrow spectrum ratio suggests that consumption of antibiotics from one subgroup was effectively replaced by the consumption of antibiotics from another subgroup.^29^

Although the sulphonamide and trimethoprim subgroup was the second-most consumed antibiotic subgroup, the CAGR declined by 7.7% from 2014 to 2018. This decline could be attributed to the South African Health Ministry’s implementation of the HIV Universal Test and Treat (UTT) programme,^30^ in accordance with international guidelines’ recommendations in September 2016.^31^ Sulphamethoxazole and trimethoprim prophylaxis was recommended in 2014 international guidelines for patients with severe or advanced HIV infection (WHO stages 3 or 4)^32^ and a CD4^+^ cell level of fewer than 200 cells/mm, according to the latest (2020) Southern African antiretroviral therapy guidelines for adults.^33^ Moreover, adults with HIV infection who are clinically stable on antiretroviral therapy and show immunological recovery and viral suppression may discontinue SMX-TMP prophylaxis.^32,33^ The number of clinically deteriorating patients who met the early criteria for SMX-TMP prophylaxis may have been reduced by the implementation of the UTT programme.^30^ The UTT programme encourages antiretroviral therapy initiation regardless of CD4^+^ cell count or WHO clinical staging towards achieving a 90% viral suppression (enabling discontinuation of SMX-TMP).^31^

In this study, cephalosporins’ fluctuating consumption can be attributed to possible stock shortages. A 2018 survey of South African public sector pharmacists revealed cephalosporins as the second-most frequently out-of-stock antibiotic, after macrolides.^24^ Antibiotic shortages do not only result in limited accessibility but also contribute to inappropriate use, which perpetuates the development and spread of ABR.^34^ Given the STG’s extensive list of indications for cephalosporins, its unavailability may result in serious adverse patient outcomes.^25^ Tetracyclines’ decrease to zero distribution by 2018 can be ascribed to changes in South African sexually transmitted infection management guidelines, which now mostly recommend using macrolides and cephalosporins such as azithromycin and ceftriaxone, respectively, over tetracyclines such as doxycycline.^35,36^ Furthermore, malaria is endemic in the Limpopo province,^37^ South Africa. Since 2017, the new malaria treatment guidelines recommended artemether–lumefantrine and artesunate over quinine and doxycycline.^38^ This could have decreased tetracycline use in the present study.

Azithromycin, a long-acting broad-spectrum macrolide, was introduced in the South African STG for the treatment of sexually transmitted infections (*Chlamydia trachomatis, Haemophilus ducreyi* and *Neisseria gonorrhoea*) because of high quinolone resistance, respiratory infections (i.e. bacterial pneumonia in penicillin allergy), gastrointestinal tract (i.e. cholera) and enteric fever (i.e. typhoid fever)^25,35,36^ may have driven the macrolides’ highest CAGR (18.5%) during the study period (2014–2018) compared with the other antibiotic subgroups. However, there was intramacrolide subgroup variability, with long-acting broad-spectrum azithromycin consumption increasing at a CAGR of 53.4% at the detriment of intermediate-acting broad-spectrum macrolide (i.e. clarithromycin, CAGR of 0.0%, Table 2) and short-acting narrow-spectrum macrolides (i.e. erythromycin, CAGR decreased by 100.0%, Table 2).^39^ Azithromycin was preferred over other macrolides (i.e. erythromycin and clarithromycin) becausde of its pharmacokinetics (long-acting, allowing once-daily dosing, which may improve patient adherence to therapy), tolerability (fewer gastrointestinal tract side effects compared with erythromycin), high intracellular concentrations and potency against enteric bacteria such as *Salmonella* species and the absence of drug–drug interactions via the cytochrome P450 drug metabolism complex.^40^

Some limitations that could have led to underreporting of ABC need to be taken into account in interpreting the results of this study. Firstly, some antibiotics had to be excluded from the analysis because of the lack of DDD allocation. Secondly, the ABC sales data did not represent the private sector and excluded some public healthcare settings. These findings can, therefore, not be generalised to the total population of the Limpopo province. Furthermore, the ABC analysis was not disaggregated by healthcare level because of data access limits; therefore, it was impossible to evaluate the existence of variability in ABC between the community (primary care) and hospital sector and seasonality. Finally, because of unavailability of sales data beyond 2018 as a result of database changes and access restrictions, only data from 2014 to 2018 were used; more recent data may show a different ABC pattern.

Despite these limitations, the study’s strength is that it is, to authors’ knowledge, the first study to describe ABC and therefore establishes a benchmark (although probably underestimated) for monitoring and evaluating future ASP policies and actions in the public sector of the Limpopo province. Public sector health facilities are the most convenient and first point of contact for healthcare to the general population of South Africa, covering health services for more than 90% of the province’s population.^13,14^

Recommendations on policy, practice and research include the following: firstly, the Limpopo Provincial Health Authority should consider implementing routine ABC surveillance reports using an internationally accepted methodology. Secondly, future research needs to ensure that ABC central warehouse data are integrated with data from direct procurement by specific healthcare facilities and data from private sector institutions and community pharmacies. Thirdly, future ABC analysis should consider determining seasonality and consumption variation between community and hospital healthcare sectors. Fourthly, the ABC surveillance data describe ABC at an ecological level and only serve as a proxy for actual antibiotic use; therefore, patient-level surveillance data are required to assess the quality of antibiotic prescribing and use. Antibiotic prescribing point prevalence surveys can be used to do this. Finally, community surveys are important to acquire a more comprehensive status of patients’ antibiotic knowledge, attitudes and practices.

In conclusion, the study quantified and described ABC in the Limpopo province’s public sector using comparable international methodologies. Antibiotic consumption has remained stable from 2014 to 2018, while broad-spectrum ABC has increased, which is associated with the development and spread of antimicrobial resistance and requires further investigation.

## Appendix 1

**TABLE 1-A1:** Absolute antibiotic consumption at Anatomical Therapeutic Chemical level 5 in defined daily dose per 1000 inhabitants per day, mean and compound annual growth rate.

Item description	2014	2015	2016	2017	2018	Mean	s.d.	Relative ABC %	CAGR %
Amoxicillin	0.732000	1.262000	1.334000	2.145000	1.589000	1.412400	± 0.5	28.4	16.8
Sulphamethoxazole and trimethoprim	1.278000	1.587000	1.443000	1.608000	0.857000	1.354600	± 0.3	27.2	−7.7
Doxycycline	0.934000	0.600000	0.295000	-	-	0.609667	± 0.3	12.3	−100.0
Azithromycin	0.077000	0.222000	0.479000	0.848000	0.657000	0.456600	± 0.3	9.2	53.5
Phenoxymethylpenicillin	0.357000	0.460000	0.163000	0.780000	0.327000	0.417400	± 0.2	8.4	−1.7
Flucloxacillin	0.286000	0.230000	0.252000	0.445000	-	0.303250	± 0.1	6.1	−100.0
Amoxicillin and clavulanic acid	0.110000	0.148000	0.161000	0.312000	0.250000	0.196200	± 0.1	3.9	17.8
Erythromycin	0.204000	0.060000	0.002000	-	-	0.088667	± 0.1	1.8	−100.0
Ciprofloxacin	0.031000	0.047000	0.074000	0.132000	0.076000	0.072000	± 0.0	1.4	19.6
Ceftriaxone	0.009900	0.013000	0.024000	0.038000	0.013000	0.019580	± 0.0	0.4	5.6
Metronidazole	0.018000	0.016000	0.004700	0.004600	0.011000	0.010860	± 0.0	0.2	−9.4
Cefixime	0.017000	0.009100	0.000190	-	-	0.008763	± 0.0	0.2	−100.0
Moxifloxacin	0.014000	0.008300	-	0.001800	-	0.008033	± 0.0	0.2	−100.0
Benzylpenicillin	0.006400	0.002500	0.003900	0.007800	0.001400	0.004400	± 0.0	0.1	−26.2
Benzathine benzylpenicillin	0.006200	0.001700	0.001400	0.003500	0.003900	0.003340	± 0.0	0.1	−8.9
Levofloxacin	0.003500	0.001400	0.000240	0.002300	-	0.001860	± 0.0	0.0	−100.0
Ampicillin	0.007700	0.000120	0.000710	0.000025	0.000001	0.001711	± 0.0	0.0	−83.5
Ofloxacin	-	0.001500	-	-	-	0.001500	± 0.0	0.0	0.0
Kanamycin	0.001900	0.000190	0.001300	0.000470	0.000910	0.000954	± 0.0	0.0	−13.7
Gentamicin	0.000740	0.001600	0.000260	0.000410	-	0.000753	± 0.0	0.0	−100.0
Linezolid	-	0.000720	-	-	-	0.000720	± 0.0	0.0	0.0
Cloxacllin	0.000770	0.000600	0.000036	0.001200	0.000200	0.000561	± 0.0	0.0	−23.6
Clarithromycin	-	0.000440	-	0.000660	-	0.000550	± 0.0	0.0	0.0
Cefazolin	0.000430	0.000016	0.000052	0.000570	0.000850	0.000384	± 0.0	0.0	14.6
Cefoxitin	0.000170	-	-	-	-	0.000170	± 0.0	0.0	−100.0
Nitrofurantoin	-	-	-	0.000150	-	0.000150	± 0.0	0.0	0.0
Cephalexin	0.000004	0.000018	-	0.000140	0.000300	0.000116	± 0.0	0.0	133.7
Cefotaxime	0.000160	0.000002	0.00013	0.000040	-	0.000083	± 0.0	0.0	−100.0
Amikacin	0.000056	-	-	0.000071	-	0.000064	± 0.0	0.0	−100.0
Vancomycin	0.000024	0.000002	-	0.000160	-	6.213E-05	± 0.0	0.0	−100.0
Imipenem+cilastatin	0.000091	0.000051	0.000076	0.000025	0.000011	0.000051	± 0.0	0.0	−34.5
Clindamycin	0.000054	0.000068	-	0.000028	-	0.000050	± 0.0	0.0	−100.0
Cefuroxime	0.000036	-	-	-	-	0.000036	± 0.0	0.0	−100.0
Meropenem	0.000005	-	-	0.000038	0.00004	0.000028	± 0.0	0.0	49.3
Cefepime	0.000019	-	-	-	-	0.000019	± 0.0	0.0	−100.0
Piperacillin+tazobactam	0.000007	0.000002	-	-	0.000002	3.013E-06	± 0.0	0.0	−48.1
Ceftazidime	-	-	0.000002	0.000002	-	0.000002	± 0.0	0.0	0.0

s.d., standard deviation; ABC, antibiotic consumption; CAGR, compound annual growth rate.
